# Isotopic Radiolabeling of Crizotinib with Fluorine-18 for In Vivo Pet Imaging

**DOI:** 10.3390/ph15121568

**Published:** 2022-12-15

**Authors:** Malvika Sardana, Louise Breuil, Sébastien Goutal, Maud Goislard, Mikhail Kondrashov, Etienne Marchal, Florent L. Besson, Christophe Dugave, Gail Wrigley, Anna C. Jonson, Bertrand Kuhnast, Magnus Schou, Nicolas Tournier, Charles S. Elmore, Fabien Caillé

**Affiliations:** 1 Early Chemical Development, Pharmaceutical Sciences, Bio Pharmaceuticals R&D, AstraZeneca, 43150 Gothenburg, Sweden; 2 Université Paris-Saclay, Inserm, CNRS, CEA, Laboratoire d’Imagerie Biomédicale Multimodale Paris-Saclay (BioMaps), 91401 Orsay, France; 3 Department of Clinical Neuroscience, Centre for Psychiatry Research, Karolinska Institutet and Stockholm County Council, SE-171 76 Stockholm, Sweden; 4 Université Paris-Saclay, Service de Chimie Bio-organique et Marquage (SCBM), CEA/DRF/JOLIOT, 91191 Gif-sur-Yvette, France; 5 Medicinal Chemistry, Oncology R&D, AstraZeneca, Cambridge CB2 0AA, UK; 6 AZ PET Science Centre at Karolinska Institutet, Oncology R&D, AstraZeneca, 15185 Stockholm, Sweden

**Keywords:** fluorine-18, radiolabeling, crizotinib, PET imaging

## Abstract

Crizotinib is a tyrosine kinase inhibitor approved for the treatment of non-small-cell lung cancer, but it is inefficient on brain metastases. Crizotinib is a substrate of the P-glycoprotein, and non-invasive nuclear imaging can be used to assess the brain penetration of crizotinib. Positron emission tomography (PET) imaging using fluorine-18-labeled crizotinib would be a powerful tool for investigating new strategies to enhance the brain distribution of crizotinib. We have synthesized a spirocyclic hypervalent iodine precursor for the isotopic labeling of crizotinib in a 2.4% yield. Because crizotinib is an enantiomerically pure drug, a chiral separation was performed to afford the *(R)*-precursor. A two-step radiolabeling process was optimized and automated using the racemic precursor to afford [^18^F]*(R,S)*-crizotinib in 15 ± 2 radiochemical yield and 103 ± 18 GBq/µmol molar activity. The same radiolabeling process was applied to the *(R)*-precursor to afford [^18^F]*(R)*-crizotinib with comparable results. As a proof-of-concept, PET was realized in a single non-human primate to demonstrate the feasibility of [^18^F]*(R)*-crizotinib in in vivo imaging. Whole-body PET highlighted the elimination routes of crizotinib with negligible penetration in the brain (SUVmean = 0.1). This proof-of-concept paves the way for further studies using [^18^F]*(R)*-crizotinib to enhance its brain penetration depending on the P-glycoprotein function.

## 1. Introduction

Crizotinib (Xalkori^®^, Pfizer, New York, NY, USA) is an FDA-approved tyrosine kinase inhibitor (TKI) for chemotherapy against non-small-cell lung cancer (NSCLC) [1,2], the leading cause of cancer mortality worldwide [3]. In three to seven percent of NSCLC tumors, anaplastic lymphoma kinase (ALK) gene rearrangements occur and lead to the expression of a transforming fusion kinase [4] targeted by crizotinib. Unfortunately, the central nervous system (CNS) remains a sanctuary site for crizotinib, and patients have a high risk of developing brain metastases, a common event in NSCLC observed in 60% of “ALK-positive” patients, resulting in significant morbidity and poor survival prognosis [5,6,7]. It has been demonstrated that crizotinib is a substrate of the P-glycoprotein (P-gp, ABCB1) transport protein, whose overexpression on cancer cells accounts for their pharmacoresistance [8]. It is very likely that P-gp is also responsible for the poor brain penetration of crizotinib across the blood–brain barrier (BBB). Indeed, Tang and coworkers demonstrated that in the presence of a P-gp inhibitor, the brain distribution of crizotinib was improved in mice [9]. However, many differences in species exist regarding the structure and function of the BBB between rodents and humans [10,11]. On the other hand, the mechanisms and functions of the BBB are well-conserved in mammals, and the expression of efflux transporters in non-human primates (NHP) is close to that of humans [12]. Investigating the brain penetration of crizotinib in NHP would be highly relevant for the validation of innovative strategies to improve the exposure of crizotinib in sanctuary sites such as the brain and to treat NSCLC brain metastases [13]. 

Positron emission tomography (PET) is a powerful non-invasive imaging technique for studying the whole-body pharmacokinetics and tissue exposure of radiolabeled drugs, including their brain penetration [14,15,16]. As an example, TKI-PET imaging using radiolabeled erlotinib demonstrated that the brain penetration of this kinase inhibitor could be enhanced in the presence of a P-gp inhibitor in NHP [15,17]. PET imaging is generally performed with microdoses (<100 µg) of the radiotracer and might not reflect the reality of the pharmacological doses used for treatment. To avoid this pitfall, TKI-PET imaging can also be performed using pharmacological doses of the drug, demonstrating the applicability of this protocol to answer clinically relevant questions [15]. 

With the presence of a fluorine atom on its scaffold, crizotinib is a candidate for isotopic labeling with fluorine-18, i.e., radiolabeling without any modification of the chemical structure and allowing for the retention of chemical and biological properties. Moreover, fluorine-18 is one of the most valuable radioisotopes for PET imaging, with an ideal half-life (t1/2 = 109.8 min) and decay properties (ß + emission of 97%). Analogs of crizotinib have already been radiolabeled with fluorine-18 [18,19], but these approaches modify the core structure of crizotinib, thus impacting the biological properties of the molecule. Therefore, PET imaging using these radiotracers cannot be used to draw any conclusion on crizotinib interactions at the BBB. To date, crizotinib itself has never been radiolabeled with fluorine-18 due to the challenging position of fluorine on an electron-rich aromatic ring. However, [^18^F]crizotinib and PET imaging would be an interesting combination to decipher the tissue exposure of crizotinib, the role played by the P-gp at the BBB on the brain penetration of crizotinib, and to investigate means to improve this penetration to treat brain metastases. Aiming at developing a new tool to explore the brain penetration of crizotinib with PET imaging and investigating means to enhance the brain exposure, we report herein the synthesis of an original iodonium ylide precursor to achieve the challenging isotopic radiolabeling of crizotinib with fluorine-18. First, this radiolabeling was optimized and automated with a racemic precursor. Because crizotinib is an enantiomerically pure drug of the *(R)* configuration, a chiral purification was performed to isolate the *(R)*-precursor, from which the radiosynthesis of [^18^F]*(R)*-crizotinib was realized. A quality control was set up to enable the first in vivo injection of [^18^F]*(R)*-crizotinib into a healthy NHP as a proof-of-concept of the feasibility of using this radiotracer for PET imaging. 

## 2. Results and Discussion

### 2.1. Chemistry

#### 2.1.1. Synthesis of the Racemic Precursor

**Scheme 1:**
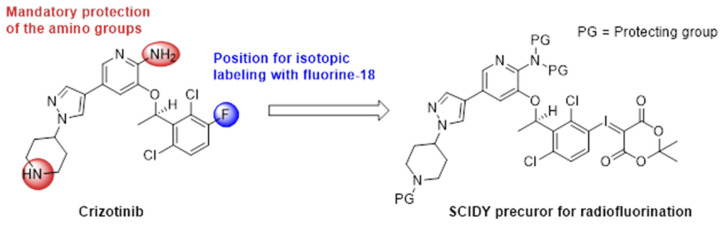
General scaffold for the SCIDY precursor for isotopic radiolabeling of crizotinib with fluorine-18.

The most efficient strategy to radiolabel crizotinib with fluorine-18 is a late-stage approach consisting of the incorporation of fluorine-18 on the aromatic core at the final step of the synthesis (Scheme 1). However, this aromatic core is electronically rich, hampering radiofluorination by standard aromatic nucleophilic substitution (SN_Ar_). Different approaches have been described in the literature to radiolabel the electronically rich aromatic core with fluorine-18, including copper-catalyzed Chan-Lam fluorination from boronic acids/esters or trialkylstannanes derivatives [20,21], Pd-catalyzed electrophilic fluorination [22], concerted SN_Ar_ from phenol derivatives [23,24], or SN_Ar_ from diazonium or iodonium precursors [25]. Among the latter, iodonium ylides and particularly spirocyclic hypervalent iodonium ylide (SCIDY) complexes are powerful precursors for regioselective radiofluorination of electron-rich and hindered aromatic cores [26,27]. Rotstein and coworkers realized the radiofluorination on a model molecule mimicking the aromatic core of crizotinib with 82% radiochemical conversion (RCC) using a SCIDY complex [27]. SCIDY can be prepared in a two-step one-pot procedure from iodine derivatives and canbe purified by standard flash chromatography, although it is sometimes difficult to separate the complexes from the iodinated starting material [28]. For radiofluorination reactions, protic moieties such as amines have to be protected [26]. All combined, a SCIDY derivative with both protected piperidine and aminopyridine moieties is a promising precursor for the radiofluorination of crizotinib (Scheme 1). Retention of the conformation of the chiral center is expected during the radiofluorination with iodonium ylides, as demonstrated by Rotstein and coworkers with the synthesis of [^18^F]fluoro-L-DOPA and [^18^F]fluoroestrone [26].

**Scheme 2:**
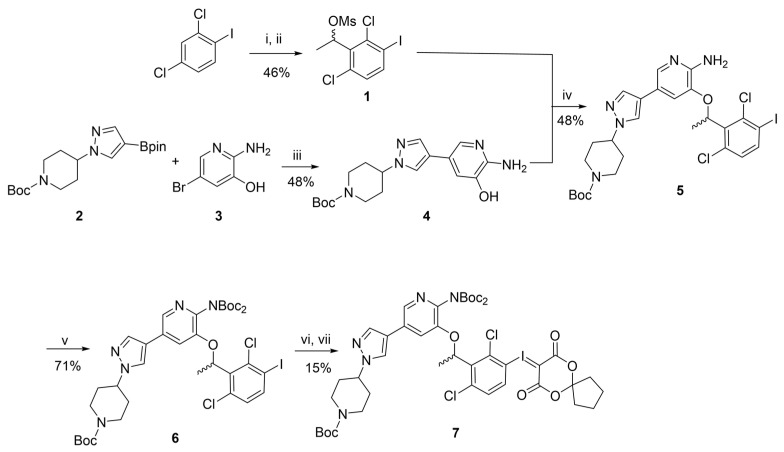
Synthesis of the racemic precursor **7**. Reagents and conditions: (i) *n*-BuLi, ^i^Pr_2_NH, acetaldehyde, THF, −78 °C, 1 h; (ii) DIPEA, MsCl, CH_2_Cl_2_, 0 °C, 1.5 h; (iii) Pd(dppf)Cl_2_, Na_2_CO_3_, H_2_O, 1,4-dioxane, 100 °C, 16 h; (iv) Cs_2_CO_3_, CH_3_CN, 60 °C, 4 h; (v) (Boc)_2_O, DIPEA, DMAP, THF, r.t., 16 h; (vi) 3,3-Dimethyldioxirane, acetone, AcOH, 0 °C, 2 h; (vii) Dioxaspiro[4.5]decane-7,9-dione, Na_2_CO_3aq_ (10%), EtOH, r.t., 2 h.

Among the different syntheses of crizotinib described in the literature, Qiang and coworkers have developed a convergent, high-yielding and cost-effective approach in which three key fragments were identified and linked together in an eight-step process [29]. Inspired by this approach, we synthesized the racemic precursor **7** (Scheme 2). Regioselective ortholithiation of 2,4-dichloro-1-iodobenzene using lithium diisopropylamine generated in situ followed by a reaction with acetaldehyde and subsequently the mesylation (Ms) of the resulting alcohol in the presence of diisopropylethylamine (DIPEA) afforded the racemic compound **1** in a 46% yield over two steps. In parallel, Suzuki coupling between boronic ester **2 [30]** and bromopyridine **3 [29]** afforded compound **4** in a 48% yield. Compounds **1** and **4** were then assembled into **5** by nucleophilic substitution in a 48% yield. Anticipating the radiofluorination for which protic groups have to be protected [26], the aminopyridine moiety was fully Boc-substituted using three equivalents of Boc_2_O and dimethylaminopyridine (DMAP) to give iodo-crizotinib derivative **6** in a 71% yield. Finally, racemic iodonium ylide **7** was synthesized in a two-step one-pot sequence. Smooth and selective oxidation in mild reaction conditions following the approach of Bravo and coworkers using dimethyldioxirane [31] was chosen to avoid side reactions on the pyridine core. Reaction of the iodoso intermediate with a spiro derivative of Meldrum’s acid as depicted by Liang et al. [28] afforded **7** with a moderate yield of 15% over the last two steps. This can be explained by the difficulty to separate compound **7** from the remaining iodine derivative **6** by flash chromatography. However, the elimination of the remaining **6** can be performed by preparative HPLC during the enantiomeric purification step depicted below. Overall and despite the low yield obtained for the ylide formation, this convergent synthesis afforded **7** on a 300 mg scale in six steps and a 2.4% overall yield. 

#### 2.1.2. Enantiomeric Purification of the Precursor

**Figure 1:**
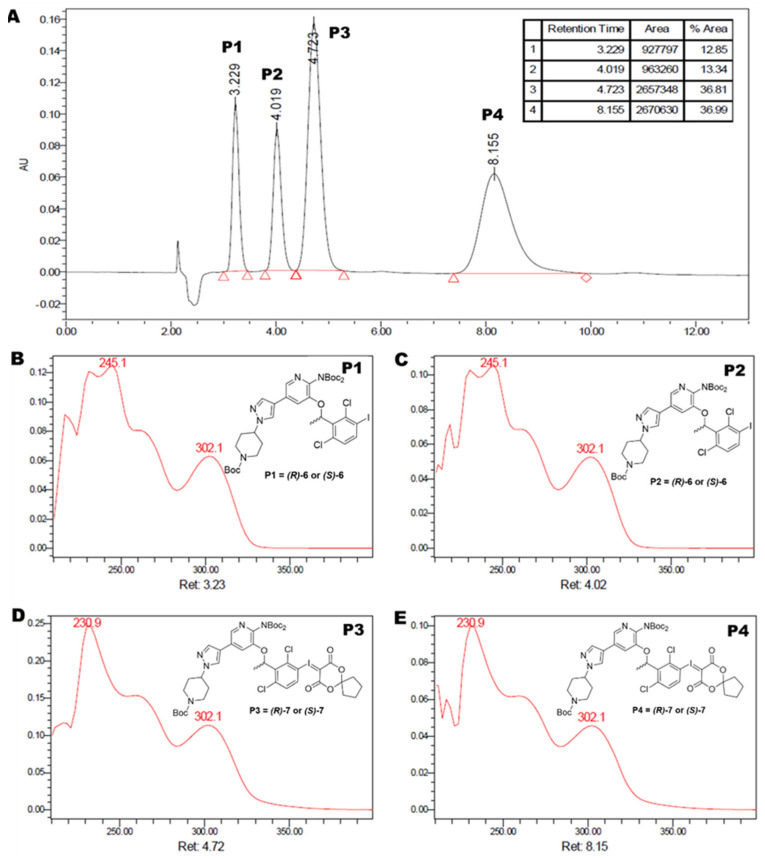
Chiral normal phase HPLC separation of **7.** (**A**) Analytical HPLC chromatogram of **7** displaying four compounds P1 to P4. Chromatographic conditions: CelluCoat^®^ (150 mm × 4.6 mm, 3 µm) column with a mixture of heptane/ethanol/trimethylamine (80/20/0.1 *v/v/v*) as eluent at a flowrate of 0.8 mL/min and UV detection 260 nm; (**B**) UV trace of the first eluted peak P1; (**C**) UV trace of the second eluted peak P2; (**D**) UV trace of the third eluted peak P3; (**E**) UV trace of the fourth eluted peak P4.

Crizotinib is an enantiomerically pure drug with the *(R)* configuration, and it has been demonstrated that *(S)* and *(R)* enantiomers display different in vivo properties, acting on different receptors [32]. Therefore, the enantiomerically pure radiotracer has to be prepared to investigate the interactions of crizotinib with the P-gp at the BBB using PET imaging, which can be achieved from the *(R)* precursor. ***(R)*-7** was obtained from the racemic precursor **7** by normal phase chiral HPLC (Figure 1A). Four compounds were observed on the HPLC chromatogram. Compounds P1 (t_R_ = 3.23 min) and P2 (t_R_ = 4.02 min) display the same UV trace, with a first absorption maximum at 245 nm and a second at 302 nm, and each represents 13% of the mixture (Figure 1B and 1C, respectively). P1 and P2 were identified by mass spectrometry (see Figures S2 and S3 in the Supplementary Materials) as the two enantiomers of the remaining iodine derivative **6**, which was difficult to separate from compound **7** by flash chromatography. Compounds P3 (t_R_ = 4.72 min) and P4 (t_R_ = 8.15 min) display the same UV trace, with a first absorption maximum at 231 nm and a second at 302 nm, and each represents 37% of the mixture (Figure 1D and 1E respectively). P3 and P4 were identified as the enantiomers of compound **7** (see Figures S4 and S5 in the Supplementary Materials). The easiest way to determine whether P3 or P4 is the *(R)* precursor is to radiolabel those precursors with fluorine-18 to obtain [^18^F]crizotinib and then perform chiral analytical HPLC using enantiomerically pure references of crizotinib.

### 2.2. Radiochemistry

#### 2.2.1. Radiofluorination Optimization

**Scheme 3:**
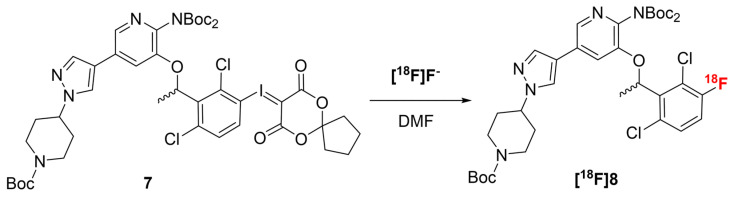
Radiofluorination of **7**. The different conditions explored are depicted in Table 1.

Optimization of the radiofluorination parameters was realized using a pure fraction of the racemic precursor **7** in order to save the enantiomerically pure precursor for preclinical studies, assuming that the results will not depend on the chirality of the precursor. Radiofluorination of SCIDY is always performed at a high temperature (T > 120 °C) [26,27]. Therefore, solvents with high boiling points such as DMF or DMSO are preferred. In the case of **7**, a better solubility was observed in DMF, which was used in the radiofluorination assays (Scheme 3). Different conditions were explored to perform the radiofluorination and optimize the conversion into **[^18^F]8** (Table 1). Radiofluorination under standard conditions using potassium carbonate and Kryptofix-222^®^ (K_222_) at 120 °C for 10 min afforded **[^18^F]8** in 18% conversion (Table 1, entry 1). This relatively low conversion rate could be explained by the poor solubility of potassium carbonate in DMF. Indeed, replacing potassium carbonate by tetraethylammonium bicarbonate resulted in a conversion of 51% (Table 1, entry 2). Increasing the temperature to 160 °C resulted in an increase in the conversion up to 64% (Table 1, entry 3). The reaction was pursued for a longer reaction time (20 min) in these conditions without improvement of the conversion (Table 1, entry 4). At high temperatures, SCIDY can be degraded by an homolytic aryl-iodine bond rupture [33], and 2,2,6,6-tetramethylpiperidine-1-oxyl (TEMPO) is often used as a radical scavenger to protect SCIDY from degradation [34,35,36]. In our case, adding TEMPO did not significantly improve the conversion into **[^18^F]8** (Table 1, entry 5).

#### 2.2.2. Deprotection Optimization

**Scheme 4:**
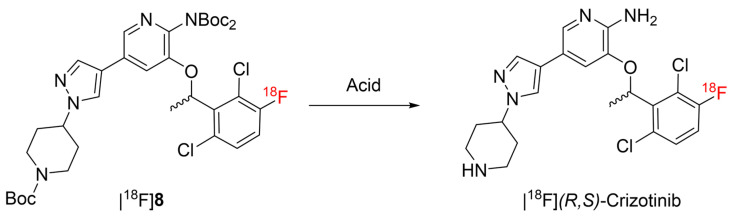
Deprotection of Boc groups on **[^18^F]8**. The different conditions explored are depicted in Table 2.

Full deprotection of Boc groups on compound **[^18^F]8** was studied to optimize the conversion into [^18^F]*(R,S)*-crizotinib (Scheme 4). Conditions explored are depicted in Table 2. Deprotection using trifluoroacetic acid (TFA) in a mixture of dioxane and water at 120 °C for 10 min was carried out after a solvent exchange realized by solid phase extraction on a Sep Pak^®^ C18 cartridge (Table 2, entry 1). We chose to add water to accelerate the reaction, as it is known to catalyze Boc deprotection at high temperatures [37]. In these conditions, only partial deprotection of compound **[^18^F]8** was observed, and conversion into [^18^F]*(R,S)*-crizotinib was only 47%. Aiming at simplifying the whole radiosynthesis process, deprotection was realized in one pot after the radiofluorination step, in DMF. Using hydrochloric acid (3M) at 120 °C for 10 min also resulted in partial deprotection and a conversion of 72% (Table 2, entry 2). Complete deprotection of all Boc groups was finally realized using HCl (3M) in DMF at 160 °C for 10 min to reach a conversion above 99% into [^18^F]*(R,S)*-crizotinib (Table 2, entry 3).

#### 2.2.3. Automated Radiosynthesis of [^18^F](*R,S*)-crizotinib

**Figure 2:**
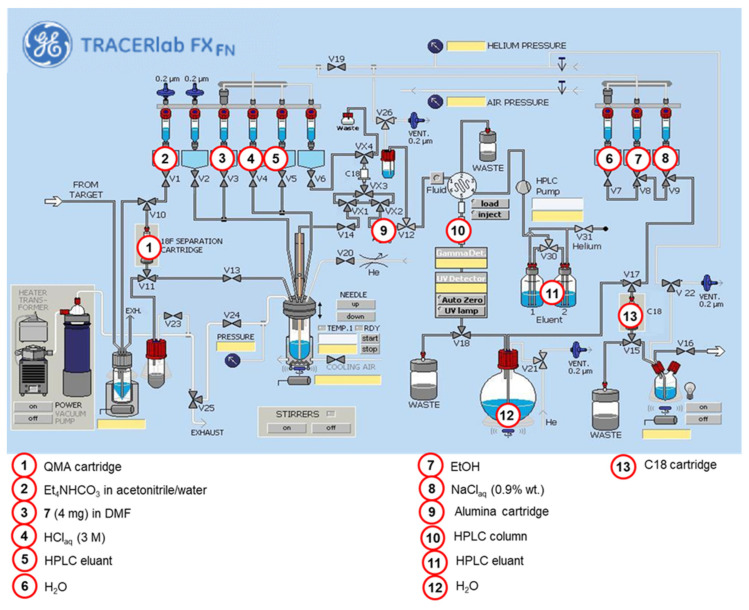
Automated radiosynthesis of [^18^F]*(R,S)*-crizotinib on a TRACERlab^®^ FX FN module.

According to the optimized conditions described above, the whole radiosynthesis process of [^18^F]*(R,S)*-crizotinib was automated using a TRACERlab^®^ FX FN module (Figure 2). [^18^F]F^−^ was recovered from the cyclotron, trapped on an anion exchange resin (QMA) cartridge, and eluted to the reactor using a solution of tetraethylammonium bicarbonate in acetonitrile and water. The Et_4_N [^18^F]F formed was dried by azeotropic distillation before radiofluorination in DMF at 160 °C for 10 min in the presence of 4 mg of precursor **7**. The aqueous hydrochloric acid solution was then added in the same reactor at 90 °C to avoid immediate vaporization of the acid, and the deprotection was carried out in one pot at 160 °C for 10 min. The crude reaction mixture was passed through an alumina cartridge to remove unreacted fluoride anions and subsequently purified by reverse-phase semi-preparative HPLC with both UV (254 nm) and gamma detection and using a mixture of acetonitrile and water as the eluent (see Figure S1 in the Supplementary Materials). The purified [^18^F]*(R,S)*-crizotinib was reformulated in a 9/1 *v/v* mixture of 0.9% NaCl_aq_ and ethanol by the mean of solid-phase extraction (SPE) on a C18 cartridge. Finally, the whole automated process afforded ready-to-inject [^18^F]*(R,S)*-crizotinib with a 15 ± 2% (*n* = 14) decay-corrected radiochemical yield (RCY) within 60 min.

#### 2.2.4. Quality Control

**Figure 3:**
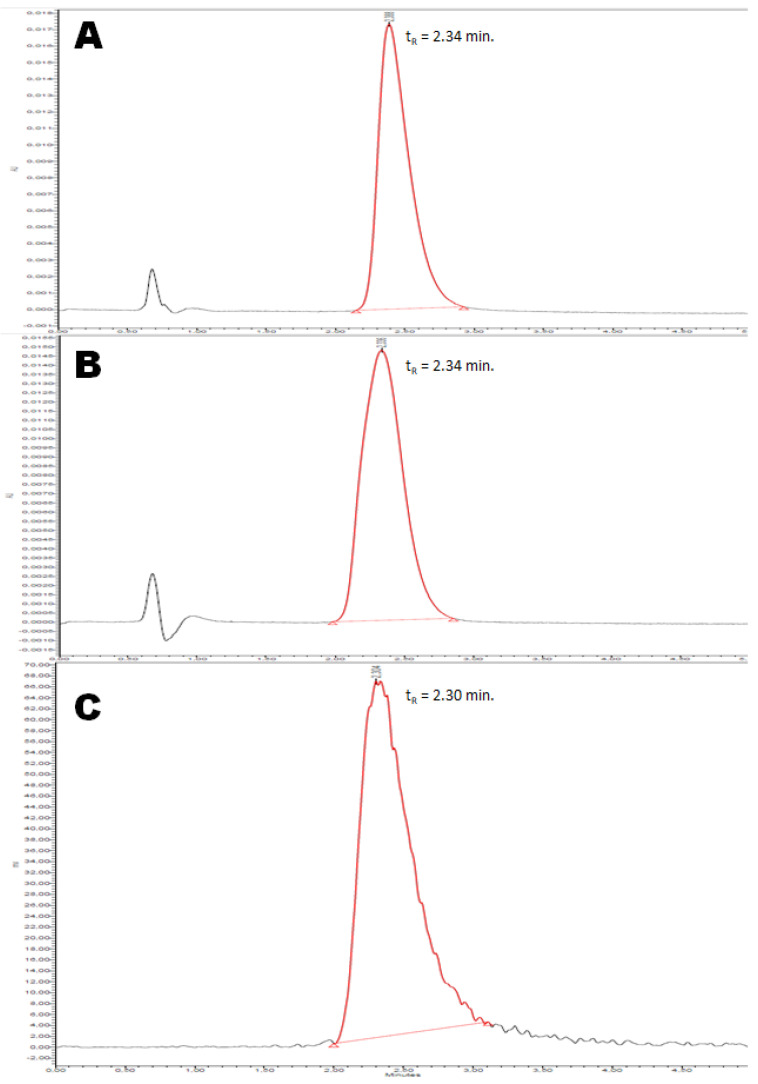
Quality control of [^18^F]*(R,S)*-crizotinib by analytical HPLC realized on a reverse-phase analytical Symmetry^®^ C18 150 × 3.9 mm, 5 μm (Waters, Milford, MA, USA) column using a mixture of H_2_O/MeOH/THF/TFA (70/20/10/0.1 *v/v/v/v*, 2 mL/min) as eluent. UV detection was performed at 333 nm. (**A**) UV chromatogram of the reference compound crizotinib; (**B**) UV chromatogram of the radiotracer [^18^F]*(R,S)*-crizotinib; (**C**) Gamma chromatogram of the radiotracer [^18^F]*(R,S)*-crizotinib.

A quality control was performed on [^18^F]*(R,S)*-crizotinib by analytical HPLC with both UV (333 nm) and gamma detection to control the identity of the radiotracer and its chemical and radiochemical purity, as well as to assess the molar activity (MA) of the radiotracer (Figure 3). The identity of the radiotracer was confirmed by the retention time of [^18^F]*(R,S)*-crizotinib (t_R_ = 2.34 min, Figure 3B), which was within the range t_R_^ref^ ± 10% compared to the retention time of the reference (t_R_^ref^ = 2.34 min, Figure 3A). [^18^F]*(R,S)*-crizotinib was obtained with both a chemical and radiochemical purity above 99% (Figure 3B and Figure 3C, respectively). [^18^F]*(R,S)*-crizotinib was obtained with a MA of 103 ± 18 GBq/µmol (*n* = 14) calculated with the help of a calibration curve. Altogether, the quality control of [^18^F]*(R,S)*-crizotinib demonstrated that the radiotracer would be suitable for in vivo injection.

#### 2.2.5. Radiosynthesis of Enantiomerically Pure [^18^F](*R*)-crizotinib

**Figure 4:**
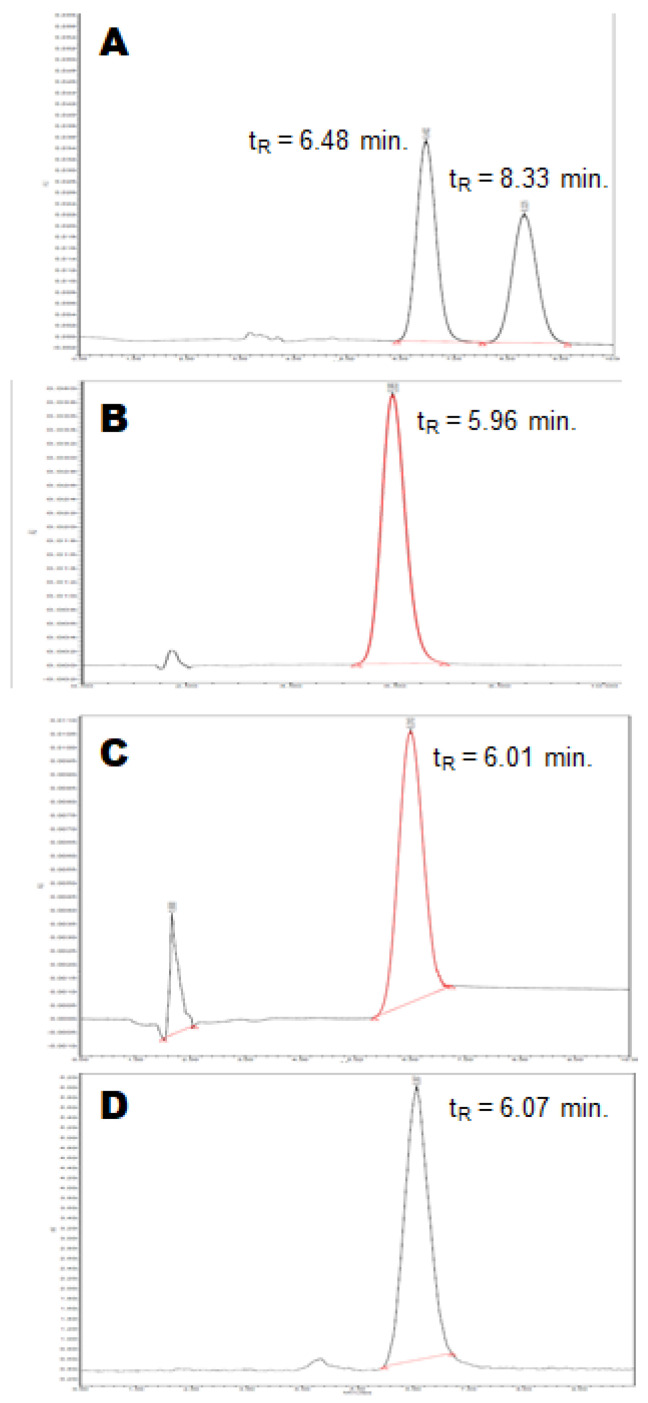
Quality control of [^18^F]*(R)*-crizotinib by analytical normal-phase chiral HPLC using a Chiralpak-AD 250 × 4.6 mm 5 µm column (Chiral Technologies, Illkirch-Graffenstaden, France) and a mixture of heptane/^i^PrOH/MeOH/*N,N*-diisopropylamine (40/30/30/0.5 *v/v/v/v*, 1 mL/min) as eluent. UV detection was performed at 333 nm. (**A**) UV chromatogram of the racemic reference; (**B**) UV chromatogram of the *(R)*-crizotinib; (**C**) UV chromatogram of the radiotracer [^18^F]*(R)*-crizotinib; (**D**) Gamma chromatogram of the radiotracer [^18^F]*(R)*-crizotinib.

We randomly chose compound P3 over P4 as the precursor to perform an automated radiosynthesis process in the exact same conditions as depicted above for the racemic precursor. The isolated radioactive compound was submitted to quality control on analytical normal phase chiral HPLC and comparted to both racemic and enantiomerically pure crizotinib references (Figure 4). The presence of two peaks at 6.48 and 8.33 min on the racemic reference chromatogram demonstrated that we were able to distinguish both enantiomers under those conditions (Figure 4A). The *(R)* enantiomer of crizotinib was identified as the peak at 5.96 min using the enantiomerically pure reference (Figure 4B). The radioactive compound synthesized from P3 showed a retention time in those conditions comparable to the *(R)*-crizotinib reference (t_R_ = 6.01 min, Figure 4C,D), demonstrating that P3 was actually the *(R)* precursor ***(R)*-7**. [^18^F]*(R)*-crizotinib was synthesized in 13 ± 3% RCY (*n* = 4) within 60 min with a MA of 112 ± 24 GBq/µmol, and results were comparable to those obtained with the racemic precursor. [^18^F]*(R)*-crizotinib was obtained with above 99% chemical and radiochemical purity (Figure 4C and Figure 4D, respectively). Despite the high temperature used in the radiosynthesis process, no racemization was observed, and [^18^F]*(R)*-crizotinib was obtained with an enantiomeric excess (ee) above 99%.

### 2.3. PET Imaging

**Figure 5:**
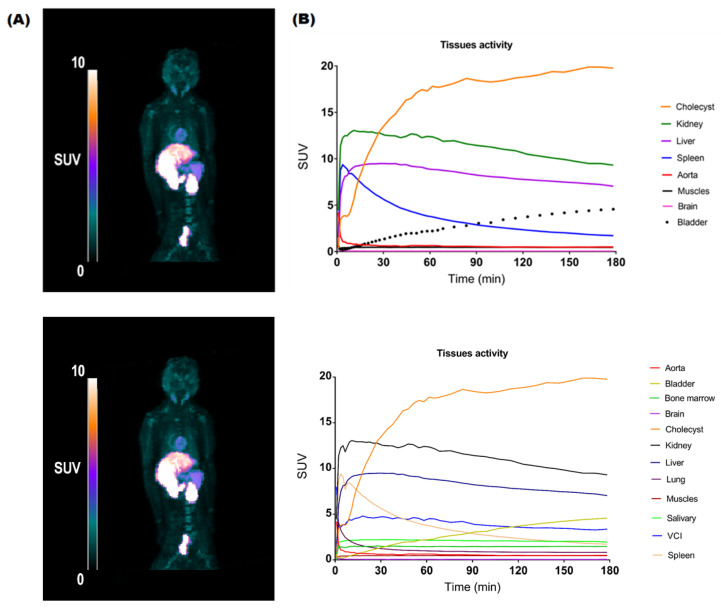
Proof-of-concept of TKI-PET imaging with [^18^F]*(R)*-crizotinib (34.8 MBq/kg) in a healthy macaque. (**A**) Whole-body PET-CT imaging at 60 min p.i.; (**B**) time-activity curves of [^18^F]*(R)*-crizotinib in blood and major organs.

To demonstrate the feasibility of in vivo NHP PET imaging, [^18^F]*(R)*-crizotinib (34.8 MBq/kg) was injected into a healthy macaque, and a whole-body dynamic PET acquisition was performed over 180 min (Figure 5A). Time-activity curves (TACs, SUV versus time) were generated in selected organs (Figure 5B). [^18^F]*(R)*-crizotinib distributed rapidly in the organs and was slowly eliminated mainly by the cholecyst route (SUV_180min_ = 19.8) and, to a lesser extent, by the liver and the kidneys (SUV_180 min_ = 7.0 and 10.6, respectively). These findings are in accordance with the known elimination routes of crizotinib in patients after oral administration of [^14^C]crizotinib [38]. A rapid washout was observed in other organs, such as the lungs (SUV_mean_ = 1.4) and muscles (SUV_mean_ = 0.5). As expected, [^18^F]*(R)*-crizotinib poorly penetrated the brain (SUV_mean_ = 0.1). This first in vivo TKI-PET imaging experiment using [^18^F]*(R)*-crizotinib paves the way for further studies using this radiotracer to evaluate strategies to improve the brain penetration of crizotinib, as previously exemplified for erlotinib in macaques [17] and humans [39].

## 3. Materials and Methods

### 3.1. Chemistry

#### 3.1.1. Chemicals

All reagents and solvents were purchased from commercial suppliers and used as received. All reactions were carried out under a nitrogen atmosphere and monitored by thin-layer chromatography on silica glass plates (60F-254, Merck, Kenilworth, NJ, USA) with UV light (254 nm), iodine vapor, or potassium permanganate as visualization agents. Crude reaction mixtures were purified by flash chromatography using prepacked SNAP columns (Biotage, Strasbourg, France) and an automated flash system with UV detection (Biotage, Strasbourg, France). ^1^H and ^13^C NMR spectra were recorded on a Bruker Advance 400 MHz apparatus using DMSO-d_6_ or CDCl_3_ as the solvent. The chemical shifts (δ) are reported in ppm (s, d, t, q, and b for singlet, doublet, triplet, quadruplet, and broad signal, respectively) and referenced with the residual solvent chemical shift. Ultra-performance liquid chromatography–mass spectroscopy (UPLC-MS) was performed on a Shimadzu LC20AD XR device equipped with a Kinetex XB-C18 column of 50 mm × 3.0 mm, 2.6 µm (Phenomenex, Le Pecq, France). A gradient of water with 0.05% of TFA and acetonitrile (5–100% of CH_3_CN for 0–1.2 min, then hold at 100% from 1.2–2.0 min) at a flowrate of 1.5 mL/min were used. Mass spectroscopy was performed with a Shimadzu LCMS2020 device equipped with an electron spray ionization (ESI) chamber. Spectra were recorded between 100 and 1000 *m/z*. High-resolution mass spectrometry (HRMS) analysis was performed by the Small Molecule Mass Spectrometry platform of ICOA (Orléans, France) by electrospray with a positive (ESI+) ionization mode.

**1-(2,6-dichloro-3-iodophenyl)ethyl methanesulfonate (1).** To a cooled (−30 °C) solution of diisopropylamine (8.2 g, 1.1 equiv.) in THF (100 mL) *n*-butyllithium (2.5 M, 31 mL, 1.1 equiv.) was added dropwise, and the solution was stirred at −30 °C for 1 h before addition dropwise into a solution of 2,4-dichloro-1-iodobenzene (20 g, 1.0 equiv.) in THF (200 mL) at −78 °C. The resulting solution was stirred for 2 h at −78 °C. Acetaldehyde (6.8 g, 2.1 equiv.) was added dropwise, and the resulting solution was stirred for 1 h at −78 °C. The reaction was quenched by the addition of an aqueous solution of ammonium chloride (100 mL). The resulting solution was extracted with ethyl acetate (2 × 200 mL). The combined organic phases were washed with a saturated solution of sodium chloride (100 mL), dried over anhydrous sodium sulfate, and concentrated under a vacuum. The residue was purified by flash chromatography on silica gel (ethyl acetate/petroleum ether) to afford the alcohol intermediate (16 g, 69%) as a yellow oil. To a cooled (0 °C) solution of this intermediate (15 g, 1.0 equiv.) in dichloromethane (200 mL), diisopropylethylamine (12.2 g, 2.0 equiv.) and methanesulfonyl chloride (7.0 g, 1.3 equiv.) were added. The resulting solution was stirred for 1.5 h at 0 °C and diluted with dichloromethane (100 mL). The organic phase was washed with a saturated solution of sodium chloride (2 × 100 mL), dried over sodium sulfate, and concentrated under a vacuum. The residue was purified by flash chromatography on silica gel (acetate/petroleum ether) to afford compound **1** (12.6 g, 67%) as an off-white solid (12.6 g, 67%). ^1^H-NMR (400 MHz, CDCl_3_) δ 7.84–7.81 (d, J = 8.4 Hz, 1H), 7.11–7.09 (d, J = 8.4 Hz, 1H), 6.57–6.50 (m, 1H), 2.92–2.88 (s, 3H), 1.85–1.80 (m, 3H) ppm.

***Tert*-butyl 4-[4-(6-amino-5-hydroxypyridin-3-yl)-1H-pyrazol-1-yl]piperidine-1-carboxylate (4)**. To a solution of compound **2 [40]** (9.8 g, 1.0 equiv.) and **3 [29]** (5.9 g, 1.2 equiv), in 1,4-dioxane (200 mL) and water (20 mL), Pd(dppf)Cl_2_ (2.0 g, 0.1 equiv.) and sodium carbonate (5.5 g, 2.0 equiv.) were added. The solution was stirred for 16 h at 100 °C. Upon cooling to room temperature, the residue was purified by flash chromatography on silica gel (dichloromethane/methanol) to afford compound **4** (4.5 g, 48%) as a light-brown solid. ^1^H-NMR (400 MHz, DMSO-d_6_) δ 8.12 (s, 1H), 7.71 (d, J = 17.4 Hz, 2H), 7.17 (s, 1H), 6.65–6.05 (b, 1H), 4.38–4.31 (b, 1H), 4.04 (d, J = 12.3 Hz, 2H), 3.01–2.89 (b, 2H), 2.03–1.99 (b, 2H), 1.91–1.84 (b, 2H), 1.42 (s, 9H) ppm. LC-MS: t_R_ = 0.786 min. 360 *m/z* [M + H]^+^.

***Tert*-butyl 4-(4-[6-amino-5-[1-(2,6-dichloro-3-iodophenyl)ethoxy]pyridin-3-yl]-1H-pyrazol-1-yl)piperidine-1-carboxylate (5)**. To a solution of **4** (4.4 g, 1.0 equiv.) and **1** (4.8 g, 1.00 equiv.) in acetonitrile (120 mL), cesium carbonate (12.0 g, 3.0 equiv.) was added. The solution was stirred for 4 h at 60 °C. Upon cooling to room temperature, the residue was purified by flash chromatography on silica gel (ethyl acetate/petroleum ether) to afford compound **5** (3.9 g, 48%) as a light-brown solid. ^1^H-NMR (400 MHz, DMSO-d_6_) δ 7.95–7.91 (m, 2H), 7.75 (m, 1H), 7.52 (s, 1H), 7.27–7.24 (d, J = 8.4 Hz, 1H), 6.84–6.83 (m, 1H), 6.12–6.10 (m, 2H), 4.34–4.29 (m, 1H), 4.10–4.00 (m, 3H), 2.92 (m, 2H), 2.03–2.00 (m, 3H), 1.80–1.75 (m, 4H), 1.43 (s, 9H) ppm. LC-MS: t_R_. = 1.135 min. 658 *m/z* [M + H]^+^, 699 *m/z* [M + MeCN + H]^+^.

***Tert*-butyl 4-[4-(6-[bis[(tert-butoxy)carbonyl]amino]-5-[1-(2,6-dichloro-3-iodophenyl)ethoxy]pyridin-3-yl)-1H-pyrazol-1-yl]piperidine-1-carboxylate (6)**. To a solution of compound 5 (3.9 g, 1.0 equiv.) in THF (100 mL), diisopropylethylamine (2.3 g, 3.0 equiv.), (Boc)_2_O (3.9 g, 3.0 equiv.), and 4-dimethylaminopyridine (0.7 g, 1.0 equiv.) were added. The solution was stirred for 16 h at 25 °C. The residue was purified by flash chromatography on silica gel (ethyl acetate/petroleum ether) to afford compound 6 (3.6 g, 71%) as a white solid. ^1^H-NMR (400 MHz, DMSO-d_6_): *δ* 8.38 (s, 1H), 8.29–8.28 (m, 1H), 7.95–7.91 (m, 2H), 7.57 (s, 1H), 7.28–7.25 (m, 1H), 6.31–6.29 (m, 1H), 4.42–4.36 (m, 1H), 4.09–4.05 (m, 2H), 2.94 (m, 2H), 2.07–2.04 (m, 2H), 1.86–1.74 (m, 5H), 1.43–1.23 (m, 27H) ppm. LC-MS: t_R_. = 3.687 min. 858 *m/z* [M + H]^+^.

***Tert*-butyl 4-(4-(6-(bis(tert-butoxycarbonyl)amino)-5-(1-(2,6-dichloro-3-((7,9-dioxo-6,10-dioxaspiro[4.5]decan-8-ylidene)-l3-iodaneyl)phenyl)ethoxy)pyridin-3-yl)-1H-pyrazol-1-yl)piperidine-1-carboxylate (7)**. To a cooled (0 °C) solution of compound 6 (1.5 g, 1.0 equiv.) in acetone (16 mL) and acetic acid (4 mL), dimethyldioxirane (50 mL) was added dropwise. The resulting solution was stirred for 2 h at 0 °C and concentrated under a vacuum. The residue was diluted with ethanol (20 mL), and 6,10-dioxaspiro[4.5]decane-7,9-dione (0.6 g, 2.0 equiv.) was added. The pH of the solution was adjusted to 10 with an aqueous solution of sodium carbonate (10% *w/v*, 5 mL). The resulting solution was stirred for 2 h at room temperature, diluted with water, (100 mL) and extracted with ethyl acetate (3 × 100 mL). The combined organic layers were washed with a saturated solution of sodium chloride (2 × 100 mL), dried over sodium sulfate, and concentrated under vacuum. The residue was purified by flash chromatography on silica gel (ethyl acetate/petroleum ether) to afford compound 7 (275 mg, 15%) as an off-white solid. ^1^H-NMR (400 MHz, CDCl_3_): *δ* 8.16 (dd, J^2^ = 23 Hz, J^3^ = 2 Hz, 1H), 7.75–7.61 (b, 2H), 7.49 (d, J = 9 Hz, 1H), 7.20 (d, J = 9 Hz, 1H), 7.08–7.02 (b, 1H), 6.07–5.99 (b, 1H), 4.31–4.22 (b, 3H), 2.91–2.85 (b, 2H), 2.23–2.19 (b, 3H), 2.16–2.11 (b, 3H), 1.98–1.88 (b, 4H), 1.84–1.80 (b, 2H), 1.76 (t, J = 8 Hz, 3H), 1.46–1.41 (b, 27H) ppm. 13C-NMR (100 MHz, CDCl_3_): *δ* 154.6 (3C), 137.5, 136.7, 136.5, 136.4, 134.0, 129.9, 129.0, 124.3, 122.8, 121.3, 120.6, 118.8, 117.6, 114.7, 82.8, 82.6, 80.7, 80.1 (2C), 73.9, 59.7, 55.5, 38.4, 37.5 (2C), 32.4 (2C), 28.5 (3C), 28.1 (3C), 28.0 (3C), 23.4 (2C), 23.3 (2C), 19.1 ppm. HR-ESI(+)-MS *m/z* calcd for C_44_H_55_Cl_2_IN_5_O_11_: 1026.2314 [M + H]+, found 1026.2309. NMR and high-resolution mass spectra are presented in the Supplementary Materials (Figures S6–S8, respectively).

#### 3.1.2. Enantiomeric Purification of **7**

The racemic mixture of **7** was separated by normal phase HPLC. The semi-preparative instrument used was equipped with a 333 pump (Gilson, Villiers-le-Bel, France) and a UV 2487 detector (Waters, USA). PDR-Chiral Inc. software (PDR-Chiral Inc., West Palm Beach, FL, USA) was used for chromatographic data collection. The preparative column used was Chiralcel^®^ OD, with dimensions of 250 × 20 mm and a particle size of 5µm (Chiral Technologies, Illkirch-Graffenstaden, France). A mixture of heptane/ethanol/triethylamine (80/20/0.1 *v/v/v*) was used as an eluent at a flowrate of 18 mL/min. Each cycle, 25 mg of **7** was injected. The chromatographic peaks were detected at 250 nm.

Collected fraction were analyzed on an Acquity Arc HPLC equipped with a 2998 PDA detector, a Quaternary Solvent Manager—R, and a Column Manager -30S, all from Waters (Waters, USA), using the software Empower^®^ 3 (Waters, USA) for chromatographic data evaluation. The analytical column used was a CelluCoat^®^ 150 × 4.6 mm 3 µm column (Nouryon, Alby, Sweden), with a mixture of heptane/ethanol/trimethylamine (80/20/0.1 *v/v/v*) as the eluent at a flowrate of 0.8 mL/min. The column temperature was 25 °C, and the chromatographic peaks were detected at 260 nm.

### 3.2. Radiochemistry

#### 3.2.1. General Procedures

All reactions were carried out using a TRACERlab^®^ FX FN module (GE Healthcare, Upspala, Sweden).

No carrier-added [^18^F]fluoride ion was produced via the ^18^O(p, n)^18^F nuclear reaction by irradiation of a 2 mL [^18^O]water (>97% enriched, Rotem) target with an IBA Cyclone-18/9 (IBA) cyclotron.

Analytical HPLC was performed using a 717_plus_ Autosampler system, a 1525 binary pump, a 2996 photodiode array detector (Waters, USA), and a Flowstar LB 513 (Berthold, Thoiry, France) gamma detector. The system was operated with the Empower^®^ 3 (Waters) software. HPLC were realized on a reverse-phase analytical Symmetry^®^ C18 (150 × 3.9 mm, 5 μm, Waters) column using a mixture of H_2_O/MeOH/THF/TFA (70/20/10/0.1 *v/v/v/v*, 2 mL/min) as the eluent. UV detection was performed at 333 nm. The chemical identification of the peak was assessed by comparing the retention time of the radiotracer with the retention time of the non-radioactive reference (t_R_^ref^). For acceptance, the retention time must be within the t_R_^ref^ ± 10% range. Radiochemical and chemical purities were calculated as the ratio of the area under the curve (AUC) of the radiotracer peak over the sum of the AUCs of all other peaks on gamma and UV chromatograms, respectively. The conversion of the reaction was calculated as the ratio of the decay-corrected activity of the radiotracer at the end of the synthesis over the activity of the starting material, both measured in an ionization chamber (Capintec^®^, Berthold, France).

#### 3.2.2. Optimization of the Radiofuorination of **7**

[^18^F]F^−^ (2–3 GBq) was trapped on an ion exchange resin QMA light (Waters) and eluted with a solution of either K_2_CO_3_ and Kryptofix-222 (2 mg and 12 mg, respectively) or tetraethyl ammonium bicarbonate (15 mg) in a mixture of CH_3_CN (800 µL) and H_2_O (200 µL). The resulting base was dried upon heating at 60 °C for 7 min under a vacuum and a stream of helium followed by heating at 120 °C for 5 min under a vacuum only. Upon cooling to 70 °C, a solution of **7** (4 mg) in DMF (700 µL) was added, and the mixture was heated at 120 °C or 160 °C for 10 min. For one experiment, TEMPO (1 mg) was added to the solution of **7** in DMF. Upon cooling to room temperature, the mixture was diluted with CH_3_CN (2 mL) and analyzed by TLC on aluminum pre-coated plates of silica gel 60F_254_ (VWR) using a mixture of dichloromethane/methanol 9/1 *v/v* as the eluent. TLC plates were analyzed a Mini-scan TLC imaging scanner equipped with a FC-3600 beta probe (Bioscan Inc.). TLC chromatograms were recorded using the Chromeleon^TM^ (ThermoFischer, Les Ulis, France) software (see Figures S9–S12 in the Supplementary Materials).

#### 3.2.3. Optimization of the Deprotection of **[^18^F]8** with TFA

The crude solution of **[^18^F]8** in DMF (700 µL) was diluted with water (10 mL) and passed through a Sep Pak^®^ C18 cartridge (Waters). The cartridge was rinsed with water (5 mL), and compound **[^18^F]8** was eluted with 1,4-dioxane (2 mL). The mixture was diluted with water (2 mL), and TFA (500 µL) was added. The solution was heated at 120 °C for 10 min. Upon cooling to room temperature, the mixture was diluted with CH_3_CN (2 mL) and analyzed by analytical HPLC according to the general procedure.

#### 3.2.4. Optimization of the Deprotection of **[^18^F]8** with HCl

The crude solution of **[^18^F]8** in DMF (700 µL) was cooled to 90 °C, and an aqueous solution of hydrochloric acid (3M, 300 µL) was added. The mixture was heated at either 120 °C or 160 °C for 10 min. Upon cooling to room temperature, the mixture was diluted with CH_3_CN (2 mL) and analyzed by analytical HPLC according to the general procedure.

#### 3.2.5. Automated Radiosynthesis of [^18^F](*R,S*)-Crizotinib

[^18^F]F^−^ (20–30 GBq) was trapped on an ion exchange resin QMA light (Waters), and a solution of tetraethyl ammonium bicarbonate (15 mg) in a mixture of CH_3_CN (800 µL) and H_2_O (200 µL) was eluted in the reactor. The resulting complex was dried upon heating at 60 °C for 7 min under a vacuum and a stream of helium, followed by heating at 120 °C for 5 min under a vacuum only. Upon cooling to 70 °C, a solution of **7** (4 mg) in DMF (700 µL) was added, and the mixture was heated at 160 °C for 10 min. After cooling to 90 °C, an aqueous solution of hydrochloric acid (3M, 300 µL) was added. The mixture was heated at 160 °C for 10 min. Upon cooling to room temperature, the mixture was diluted with a mixture of H_2_O/MeOH/THF/TFA 70/20/10/0.1 *v/v/v/v* (3 mL), and the crude was purified by reverse-phase semi-preparative HPLC (Waters Symmetry^®^ C18 7.8 × 300 mm, 7 μm) with a 515 HPLC Pump (Waters, USA) using a mixture of H_2_O/MeOH/THF/TFA (70/20/10/0.1 *v/v/v/v*, 5 mL/min) as the eluent. UV detection (K2501, Knauer, Berlin, Germany) was performed at 254 nm. The purified compound was diluted with water (20 mL) and passed through a Sep-Pak^®^ C18 cartridge (Waters). The cartridge was rinsed with water (10 mL) and eluted with ethanol (2 mL), and the final compound was diluted with saline (0.9% *w/v*, 8 mL). Ready-to-inject [^18^F]*(R,S)*-crizotinib (2–3 GBq) was obtained in 15 ± 2% (*n* = 14) non-decay corrected radiochemical yield within 60 min.

#### 3.2.6. Quality Control of [^18^F](*R,S*)-Crizotinib

The quality control was performed by reverse-phase analytical HPLC according to the general procedure described above. Molar activity (MA) was calculated as the ratio of the activity of the collected peak of [^18^F]*(R,S)*-crizotinib measured in an ionization chamber (Capintec^®^, Thoiry, Berthold) over the molar quantity of crizotinib determined using a calibration curve. MA is calculated as the mean value of three consecutive runs.

#### 3.2.7. Automated Radiosynthesis of [^18^F](*R*)-Crizotinib

The radiosynthesis of [^18^F]*(R)*-crizotinib was realized in the exact same conditions as for the radiosynthesis of [^18^F]*(R,S)*-crizotinib starting from [^18^F]F^−^ (20–30 GBq) and using ***(R)*-7** (4 mg) as the precursor. Ready-to-inject [^18^F]*(R)*-crizotinib (1.9–2.9 GBq) was obtained in 13 ± 3% (*n* = 4) non-decay corrected radiochemical yield within 60 min.

#### 3.2.8. Quality Control of [^18^F](*R*)-Crizotinib

The quality control was performed using a 717_plus_ Autosampler system, a 1525 binary pump, a 2996 photodiode array detector (Waters, USA), and a Flowstar LB 513 (Berthold, France) gamma detector. The system was operated with the Empower^®^ 3 (Waters) software. Analytical HPLC was performed using a normal phase Chiralpak-AD 250 × 4.6 mm 5 µm column (Chiral Technologies, France) and a mixture of heptane/^i^PrOH/MeOH/*N,N*-diisopropylamine (40/30/30/0.5 *v/v/v/v*, 1 mL/min) as the eluent. UV detection was performed at 333 nm. Molar activity was calculated as the ratio of the activity of the collected peak of [^18^F]*(R)*-crizotinib measured in an ionization chamber (Capintec^®^, Berthold) over the molar quantity of crizotinib determined using a calibration curve. MA is calculated as the mean value of three consecutive runs.

### 3.3. PET Imaging

#### 3.3.1. Animal

An experiment was conducted on one young adult male cynomolgus monkey (*Macaca fascicularis*, 5.5 kg, 4.4 years). The animal use procedure was in accordance with the recommendations of the European regulations (EU Directive 2010/63) and approved by the local ethical committee (CETEA n°44) and the French Ministry of Education and Research (APAFIS#19200-2017121312309702).

#### 3.3.2. PET Imaging

Whole-body PET imaging was performed on a hybrid PET/CT device (Biograph Horizon, Siemens Healthineers, Germany) under standard anesthesia with monitoring procedures [41]. The imaging protocol combines a non-contrast low-dose CT scan for attenuation correction and anatomical landmarks, with a subsequent PET dynamic acquisition of 180 min. Data acquisition started with the i.v. bolus injection of [^18^F]*(R)*-crizotinib (34.8 MBq/kg, 0.29 µg/kg). All the dynamic PET images were reconstructed using a 2D iterative model (TOF-OSEM-2D) with time of flight and point spread function modeling, and with random, dead time, scatter, decay, and attenuation corrections, with post-filtering.

#### 3.3.3. PET Data Analysis

The following organs of interest were semi-automatically segmented on the fused PET/CT data using ITK-SNAP (version 3.8, http://www.itksnap.org (accessed on 25 May 2021)) [42]: the brain, aorta, cholecyst, kidney, liver, bladder, muscle, and spleen. These segmentation masks and the dynamic PET data were then transferred into PMOD software (PMOD Technologies Ltd., Zurich, Switzerland); the dynamic PET data, converted into standardized uptake values (SUV, unitless), were finally masked to generate the mean time-activity curve of each organ of interest. 

## 4. Conclusions

Crizotinib is a TKI used in the treatment of NSCLC that does not cross the BBB and shows poor efficiency on brain metastases. Because PET imaging using [^18^F]*(R)*-crizotinib would be a powerful asset to investigate the brain penetration of crizotinib depending on the P-gp function, we have realized the first fluorine-18 isotopic radiolabeling of crizotinib. An original SCIDY precursor was synthesized in six steps and a 2.4% overall yield to circumvent the difficulty of the standard Sn_Ar_ approach on this scaffold. A two-step radiosynthesis approach including radiofluorination and deprotection was optimized and automated to produce the racemic version [^18^F]*(R,S)*-crizotinib in 15 ± 2% RCY and 103 ± 18 GBq/µmol MA. Because crizotinib is an enantiomerically pure drug, the *(R)*-precursor was obtained by chiral separation and used to obtain [^18^F]*(R)*-crizotinib in 13 ± 3% RCY and 112 ± 24 GBq/µmol MA. As a proof-of-concept, PET imaging was realized in one healthy macaque and displayed the expected biodistribution and known elimination routes of crizotinib, confirming that the radiotracer does not penetrate the brain. Since no conclusion can be drawn from only one animal in a PET imaging study, further experiments are needed to explore and enhance the brain penetration of crizotinib.

## Figures and Tables

**Table 1:** Radiofluorination conditions explored for the synthesis of **[^18^F]8**.

Entry ^1^	Base	Conditions ^2^	Conversion ^3^
**1**	K_2_CO_3_ + K_222_	DMF, 120 °C, 10 min.	18%
**2**	Et_4_NHCO_3_	DMF, 120 °C, 10 min.	51%
**3**	Et_4_NHCO_3_	DMF, 160 °C, 10 min.	64%
**4**	Et_4_NHCO_3_	DMF, 160 °C, 20 min.	64%
**5**	Et_4_NHCO_3_ + TEMPO (1 mg)	DMF, 160 °C, 10 min.	48%

^1^ Each experiment was performed in duplicate. ^2^ Radiofluorinations were performed using 4 mg of **7**. ^3^ Conversion rates were measured by thin-layer chromatography.

**Table 2:** Reaction conditions explored for the deprotection of Boc groups on **[^18^F]8**.

Entry ^1^	Acid	Conditions	Conversion ^2^
**1**	TFA	Dioxane/H_2_O (1/1 *v/v*) 120 °C, 10 min.	47%
**2**	HCl 3M	DMF 120 °C, 10 min.	72%
**3**	HCl 3M	DMF 160 °C, 10 min.	>99%

^1^ Each experiment was performed in duplicate. ^2^ Conversion rates were measured by analytical HPLC.

## Data Availability

Not applicable.

## Supplementary Materials

The following supporting information can be downloaded at: https://www.mdpi.com/article/10.3390/ph15121568/s1, Figure S1: Semi-preparative HPLC purification of [^18^F]*(R,S)*-crizotinib, which is obtained at a retention time of approx. 9.2 min; Figure S2: LC-MS^+^ analysis of compound P1; Figure S3: LC-MS^+^ analysis of compound P2; Figure S4: LC-MS^+^ analysis of compound P3; Figure S5: LC-MS^+^ analysis of compound P4; Figure S6: ^1^H NMR of compound **7**; Figure S7: ^13^C NMR of compound **7**; Figure S8: HRMS analysis of compound **7**; Figure S9: TLC of the crude radiofluorination of **7** using the K [^18^F]F/K_222_ complex in DMF at 120 °C for 10 min; Figure S10: TLC of the crude radiofluorination of **7** using Et_4_N [^18^F]F in DMF at 160 °C for 10 min; Figure S11: TLC of the crude radiofluorination of **7** using Et_4_N [^18^F]F in DMF at 160 °C for 10 min; Figure S12: TLC of the crude radiofluorination of **7** using Et_4_N [^18^F]F and TEMPO (1 mg) in DMF at 160 °C for 10 min.

## References

[1] Shaw A.T., Yasothan U., Kirkpatrick P. (2011). Crizotinib. Nat. Rev. Drug Discov..

[2] Kwak E.L., Bang Y.-J., Camidge D.R., Shaw A.T., Solomon B., Maki R.G., Ou S.-H.I., Dezube B.J., Jänne P.A., Costa D.B. (2010). Anaplastic Lymphoma Kinase Inhibition in Non–Small-Cell Lung Cancer. N. Engl. J. Med..

[3] Lung Cancer—Non-Small Cell—Statistics. Cancer.Net. https://www.cancer.net/cancer-types/lung-cancer-non-small-cell/statistics.

[4] Soda M., Choi Y.L., Enomoto M., Takada S., Yamashita Y., Ishikawa S., Fujiwara S., Watanabe H., Kurashina K., Hatanaka H. (2007). Identification of the Transforming EML4-ALK Fusion Gene in Non-Small-Cell Lung Cancer. Nature.

[5] Preusser M., Berghoff A.S., Koller R., Zielinski C.C., Hainfellner J.A., Liebmann-Reindl S., Popitsch N., Geier C.B., Streubel B., Birner P. (2015). Spectrum of Gene Mutations Detected by next Generation Exome Sequencing in Brain Metastases of Lung Adenocarcinoma. Eur. J. Cancer.

[6] Shi W., Dicker A.P. (2015). CNS Metastases in Patients with Non–Small-Cell Lung Cancer and ALK Gene Rearrangement. J. Clin. Oncol..

[7] Gainor J.F., Ou S.-H.I., Logan J., Borges L.F., Shaw A.T. (2013). The Central Nervous System as a Sanctuary Site in ALK-Positive Non–Small-Cell Lung Cancer. J. Thorac. Oncol..

[8] Katayama R., Sakashita T., Yanagitani N., Ninomiya H., Horiike A., Friboulet L., Gainor J.F., Motoi N., Dobashi A., Sakata S. (2015). P-Glycoprotein Mediates Ceritinib Resistance in Anaplastic Lymphoma Kinase-Rearranged Non-Small Cell Lung Cancer. EBioMedicine.

[9] Tang S.C., Nguyen L.N., Sparidans R.W., Wagenaar E., Beijnen J.H., Schinkel A.H. (2014). Increased Oral Availability and Brain Accumulation of the ALK Inhibitor Crizotinib by Coadministration of the P-Glycoprotein (ABCB1) and Breast Cancer Resistance Protein (ABCG2) Inhibitor Elacridar. Int. J. Cancer.

[10] Deo A.K., Theil F.-P., Nicolas J.-M. (2013). Confounding Parameters in Preclinical Assessment of Blood–Brain Barrier Permeation: An Overview with Emphasis on Species Differences and Effect of Disease States. Mol. Pharm..

[11] Huttunen K.M., Terasaki T., Urtti A., Montaser A.B., Uchida Y. (2022). Pharmacoproteomics of Brain Barrier Transporters and Substrate Design for the Brain Targeted Drug Delivery. Pharm. Res..

[12] Ito K., Uchida Y., Ohtsuki S., Aizawa S., Kawakami H., Katsukura Y., Kamiie J., Terasaki T. (2011). Quantitative Membrane Protein Expression at the Blood–Brain Barrier of Adult and Younger Cynomolgus Monkeys. J. Pharm. Sci..

[13] Pandit R., Chen L., Götz J. (2020). The Blood-Brain Barrier: Physiology and Strategies for Drug Delivery. Adv. Drug Deliv. Rev..

[14] Pottier G., Marie S., Goutal S., Auvity S., Peyronneau M.-A., Stute S., Boisgard R., Dollé F., Buvat I., Caillé F. (2016). Imaging the Impact of the P-Glycoprotein (ABCB1) Function on the Brain Kinetics of Metoclopramide. J. Nucl. Med..

[15] Tournier N., Goutal S., Auvity S., Traxl A., Mairinger S., Wanek T., Helal O.-B., Buvat I., Soussan M., Caillé F. (2017). Strategies to Inhibit ABCB1- and ABCG2-Mediated Efflux Transport of Erlotinib at the Blood–Brain Barrier: A PET Study on Nonhuman Primates. J. Nucl. Med..

[16] Tournier N., Saba W., Cisternino S., Peyronneau M.-A., Damont A., Goutal S., Dubois A., Dollé F., Scherrmann J.-M., Valette H. (2013). Effects of Selected OATP and/or ABC Transporter Inhibitors on the Brain and Whole-Body Distribution of Glyburide. AAPS J..

[17] Tournier N., Goutal S., Mairinger S., Hernández-Lozano I., Filip T., Sauberer M., Caillé F., Breuil L., Stanek J., Freeman A.F. (2021). Complete Inhibition of ABCB1 and ABCG2 at the Blood–brain Barrier by Co-Infusion of Erlotinib and Tariquidar to Improve Brain Delivery of the Model ABCB1/ABCG2 Substrate [^11^C]erlotinib. J. Cereb. Blood Flow Metab..

[18] Lin Q., Zhang Y., Fu Z., Hu B., Si Z., Zhao Y., Shi H., Cheng D. (2020). Synthesis and Evaluation of ^18^F Labeled Crizotinib Derivative [^18^F]FPC as a Novel PET Probe for Imaging c-MET-Positive NSCLC Tumor. Bioorg. Med. Chem..

[19] Radaram B., Piwnica-Worms D., Alauddin M. (2018). Development of Novel Probes for PET Imaging of Lung Cancer Overexpressing Anaplastic Lymphoma Kinase: Synthesis and Radiolabeling of Crizotinib and Alectinib Analogues. J. Nucl. Med..

[20] Tredwell M., Preshlock S.M., Taylor N.J., Gruber S., Huiban M., Passchier J., Mercier J., Génicot C., Gouverneur V. (2014). A General Copper-Mediated Nucleophilic ^18^F Fluorination of Arenes. Angew. Chem..

[21] Makaravage K.J., Brooks A.F., Mossine A.V., Sanford M.S., Scott P.J.H. (2016). Copper-Mediated Radiofluorination of Arylstannanes with [^18^F]KF. Org. Lett..

[22] Lee E., Kamlet A.S., Powers D.C., Neumann C.N., Boursalian G.B., Furuya T., Choi D.C., Hooker J.M., Ritter T. (2011). A Fluoride-Derived Electrophilic Late-Stage Fluorination Reagent for PET Imaging. Science.

[23] Neumann C.N., Hooker J.M., Ritter T. (2016). Concerted Nucleophilic Aromatic Substitution with ^19^F^−^and ^18^F^−^. Nature.

[24] Beyzavi M.H., Mandal D., Strebl M.G., Neumann C.N., D’Amato E.M., Chen J., Hooker J.M., Ritter T. (2017). ^18^F-Deoxyfluorination of Phenols via Ru π-Complexes. ACS Cent. Sci..

[25] Kohlhepp S.V., Gulder T. (2016). Hypervalent iodine(III) Fluorinations of Alkenes and Diazo Compounds: New Opportunities in Fluorination Chemistry. Chem. Soc. Rev..

[26] Rotstein B.H., Stephenson N.A., Vasdev N., Liang S.H. (2014). Spirocyclic Hypervalent iodine(III)-Mediated Radiofluorination of Non-Activated and Hindered Aromatics. Nat. Commun..

[27] Rotstein B.H., Wang L., Liu R.Y., Patteson J., Kwan E.E., Vasdev N., Liang S.H. (2016). Mechanistic Studies and Radiofluorination of Structurally Diverse Pharmaceuticals with Spirocyclic iodonium(III) Ylides. Chem. Sci..

[28] Liang S.H., Wang L., Stephenson N.A., Rotstein B.H., Vasdev N. (2019). Facile ^18^F Labeling of Non-Activated Arenes via a Spirocyclic iodonium(III) Ylide Method and Its Application in the Synthesis of the mGluR5 PET Radiopharmaceutical [^18^F]FPEB. Nat. Protoc..

[29] Qian J.-Q., Yan P.-C., Che D.-Q., Zhou Q.-L., Li Y.-Q. (2014). A Novel Approach for the Synthesis of Crizotinib through the Key Chiral Alcohol Intermediate by Asymmetric Hydrogenation Using Highly Active Ir-Spiro-PAP Catalyst. Tetrahedron Lett..

[30] Liu N., Wang Y., Huang G., Ji C., Fan W., Li H., Cheng Y., Tian H. (2016). Design, Synthesis and Biological Evaluation of 1H-pyrrolo[2,3-B]pyridine and 1H-pyrazolo[3,4-B]pyridine Derivatives as c-Met Inhibitors. Bioorganic Chem..

[31] Bravo A., Fontana F., Fronza G., Minisci F., Serri A. (1995). Oxidation of Alkyl and Aryl Iodides, Phenylacetaldehyde and Alkenes by Dimethyldioxirane. Reaction Products and Mechanism. Tetrahedron Lett..

[32] Dai X., Guo G., Zou P., Cui R., Chen W., Chen X., Yin C., He W., Vinothkumar R., Yang F. (2017). (S)-Crizotinib Induces Apoptosis in Human Non-Small Cell Lung Cancer Cells by Activating ROS Independent of MTH1. J. Exp. Clin. Cancer Res..

[33] Grushin V.V. (1992). Carboranylhalonium Ions: From Striking Reactivity to a Unified Mechanistic Analysis of Polar Reactions of Diarylhalonium Compounds. Acc. Chem. Res..

[34] Lee B.C., Kim J.S., Kim B.S., Son J.Y., Hong S.K., Park H.S., Moon B.S., Jung J.H., Jeong J.M., Kim S.E. (2011). Aromatic Radiofluorination and Biological Evaluation of 2-Aryl-6-[^18^F]fluorobenzothiazoles as a Potential Positron Emission Tomography Imaging Probe for β-Amyloid Plaques. Bioorg. Med. Chem..

[35] Carroll M.A., Nairne J., Smith G., Widdowson D.A. (2007). Radical Scavengers: A Practical Solution to the Reproducibility Issue in the Fluoridation of Diaryliodonium Salts. J. Fluor. Chem..

[36] Moon B.S., Kil H.S., Park J.H., Kim J.S., Park J., Chi D.Y., Lee B.C., Kim S.E. (2011). Facile Aromatic Radiofluorination of [^18^F]flumazenil from Diaryliodonium Salts with Evaluation of Their Stability and Selectivity. Org. Biomol. Chem..

[37] Wang J., Liang Y.-L., Qu L. (2009). Boiling water-catalyzed neutral and selective *N*-Boc deprotection. Chem. Commun..

[38] Johnson T.R., Tan W., Goulet L., Smith E.B., Yamazaki S., Walker G.S., O’Gorman M.T., Bedarida G., Zou H.Y., Christensen J.G. (2015). Metabolism, Excretion and Pharmacokinetics of [^14^C]crizotinib Following Oral Administration to Healthy Subjects. Xenobiotica.

[39] Bauer M., Karch R., Wulkersdorfer B., Philippe C., Nics L., Klebermass E.-M., Weber M., Poschner S., Haslacher H., Jäger W. (2019). A Proof-of-Concept Study to Inhibit ABCG_2-_ and ABCB_1-_Mediated Efflux Transport at the Human Blood–Brain Barrier. J. Nucl. Med..

[40] De Koning P.D., McAndrew D., Moore R., Moses I.B., Boyles D.C., Kissick K., Stanchina C.L., Cuthbertson T., Kamatani A., Rahman L. (2011). Fit-for-Purpose Development of the Enabling Route to Crizotinib (PF-02341066). Org. Process Res. Dev..

[41] Auvity S., Saba W., Goutal S., Leroy C., Buvat I., Cayla J., Caillé F., Bottlaender M., Cisternino S., Tournier N. (2017). Acute Morphine Exposure Increases the Brain Distribution of [^18^F]DPA-714, a PET Biomarker of Glial Activation in Nonhuman Primates. Int. J. Neuropsychopharmacol..

[42] Yushkevich P.A., Piven J., Hazlett H.C., Smith R.G., Ho S., Gee J.C., Gerig G. (2006). User-Guided 3D Active Contour Segmentation of Anatomical Structures: Significantly Improved Efficiency and Reliability. NeuroImage.

