# Supplementary material for: Isotopic Radiolabeling of Crizotinib with Fluorine-18 for In Vivo Pet Imaging
Source: Pharmaceuticals (Basel). 2022 Dec 15;15(12):1568. doi: 10.3390/ph15121568 (PMC9782192; doi:10.3390/ph15121568)
Supplement: Supplementary file 1 [file pharmaceuticals-15-01568-s001.zip › pharmaceuticals-2071597-supplementary.pdf]

ISOTOPIC RADIOLABELING OF CRIZOTINIB WITH FLUORINE-18 FOR IN VIVO PET IMAGING

Malvika Sardana[1,2], Louise Breuil[2], Sébastien Goutal[2], Maud Goislard[2], Mikhail Kondrashov[3], Etienne Marchal[2], Florent L. Besson[2], Christophe Dugave[4], Gail Wrigley[5], Anna C. Jonson[1], Bertrand Kuhnast[2], Magnus Schou[3,6], Nicolas Tournier[2], Charles S. Elmore[1] and Fabien Caillé[2,*]

[1]Early Chemical Development, Pharmaceutical Sciences, Bio Pharmaceuticals R&D, AstraZeneca, Gothenburg, Sweden
[2]Université Paris-Saclay, Inserm, CNRS, CEA, Laboratoire d'Imagerie Biomédicale Multimodale Paris-Saclay (BioMaps), 91401 Orsay
[3]Department of Clinical Neuroscience, Centre for Psychiatry Research, Karolinska Institutet and Stockholm County Council, SE-171 76 Stockholm, Stockholm, Sweden
[4]Université Paris-Saclay, Service de Chimie Bio-organique et Marquage (SCBM), CEA/DRF/JOLIOT, 91191, Gif-sur-Yvette, France
[5]Medicinal Chemistry, Oncology R&D, AstraZeneca, Cambridge, UK
[6]AZ PET Science Centre at Karolinska Institutet, Oncology R&D, AstraZeneca, Stockholm, Sweden

[*]Corresponding author :
Fabien Caillé, PhD
CEA-Service Hospitalier Frédéric Joliot
4, place du Général Leclerc
91401 Orsay, France
fabien.caille@cea.fr
ORCID: 0000-0003-0088-7337

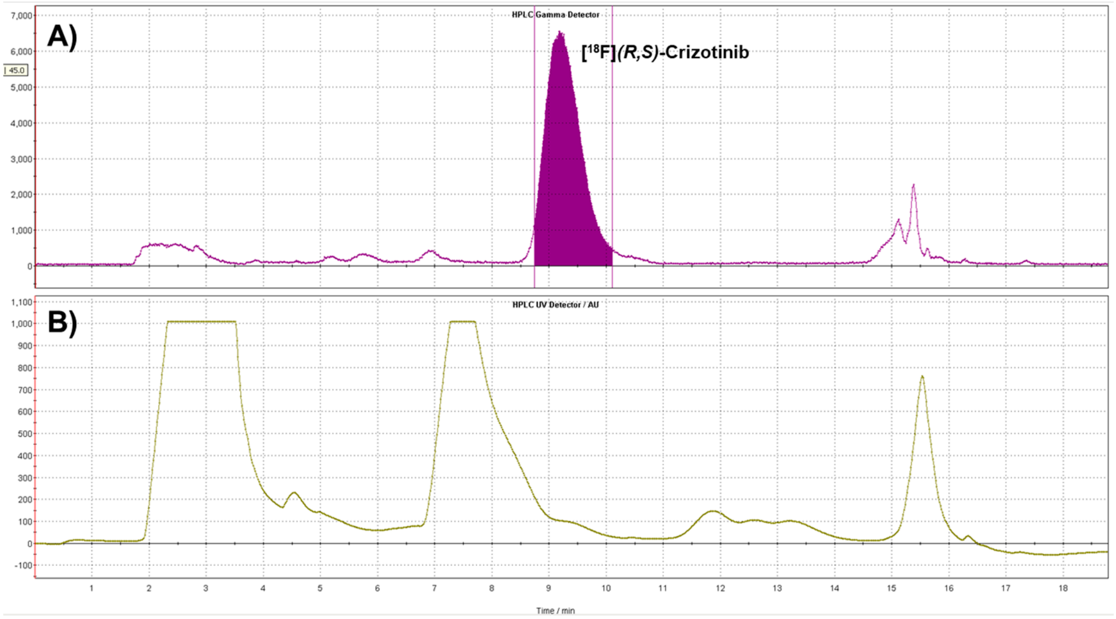

Figure S1. Semi-preparative HPLC purification of [^{18}F]*(R,S)*-crizotinib, which is obtained at a retention time of approx. 9.2 min. A) Gamma detection; B) UV detection at 254 nm.

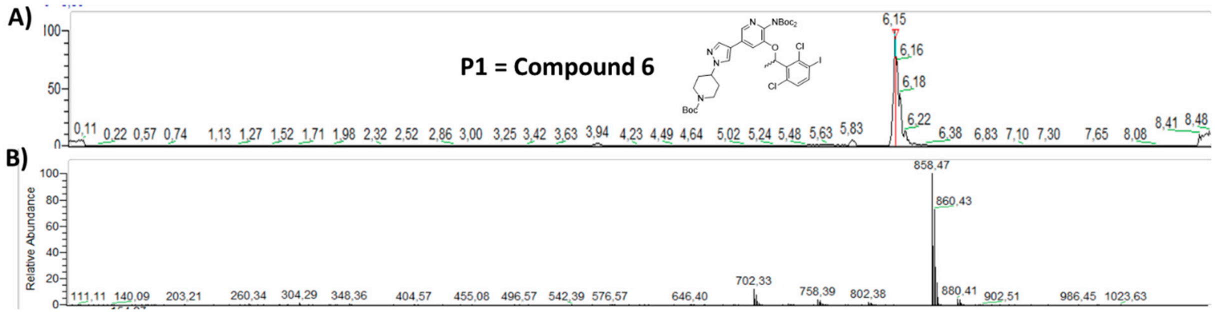

Figure S2. LC-MS$^+$ analysis of compound P1. A) LC analysis with MS$^+$ detection; B) Positive mass spectroscopy of the peak at t_R = 6.15 min.

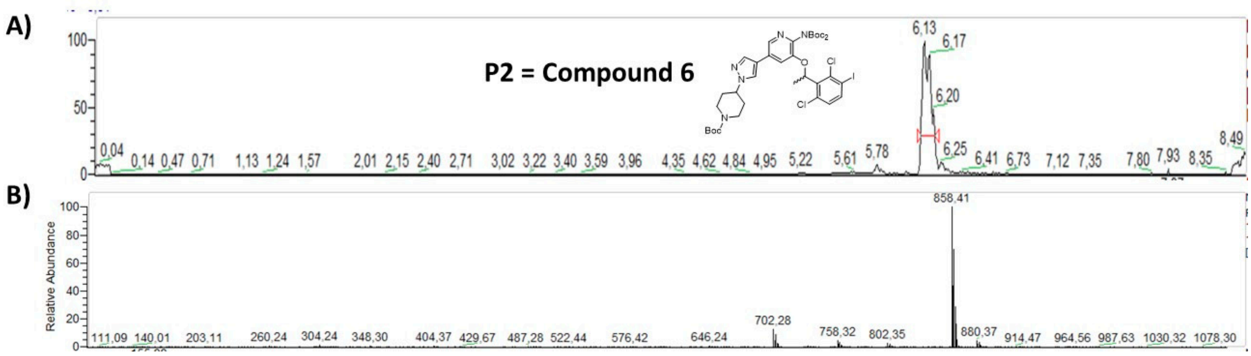

Figure S3. LC-MS$^+$ analysis of compound P2. A) LC analysis with MS$^+$ detection; B) Positive mass spectroscopy of the peak at t_R = 6.13-6.20 min.

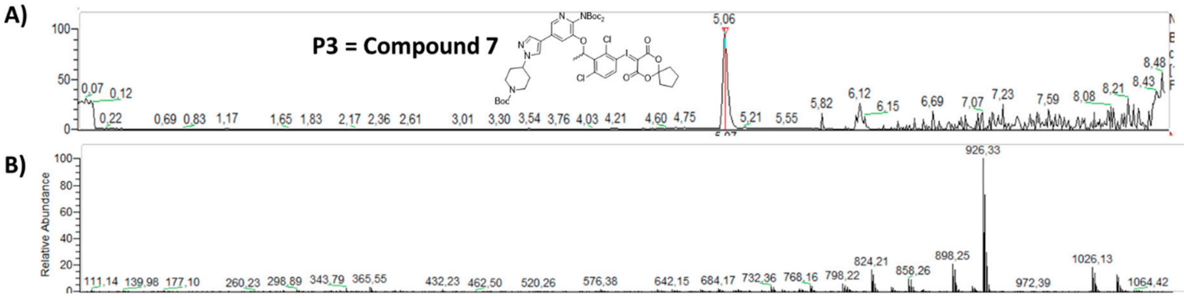

Figure S4. LC-MS$^+$ analysis of compound P3. A) LC analysis with MS$^+$ detection; B) Positive mass spectroscopy of the peak at t_R = 5.06 min.

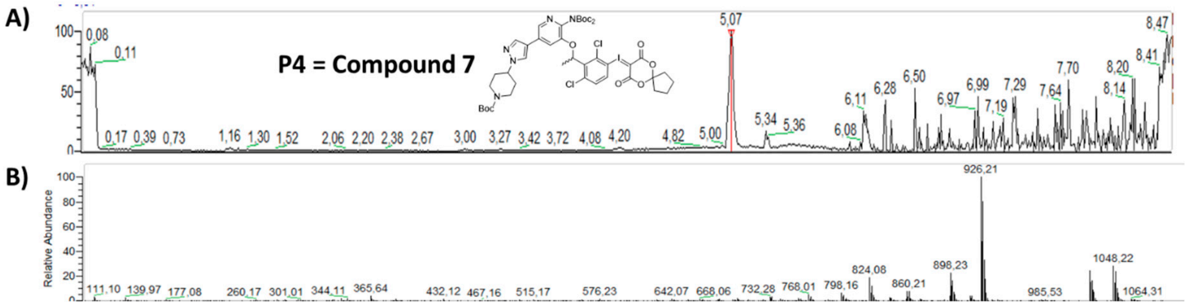

Figure S5. LC-MS$^+$ analysis of compound P4. A) LC analysis with MS$^+$ detection; B) Positive mass spectroscopy of the peak at t_R = 5.07 min.

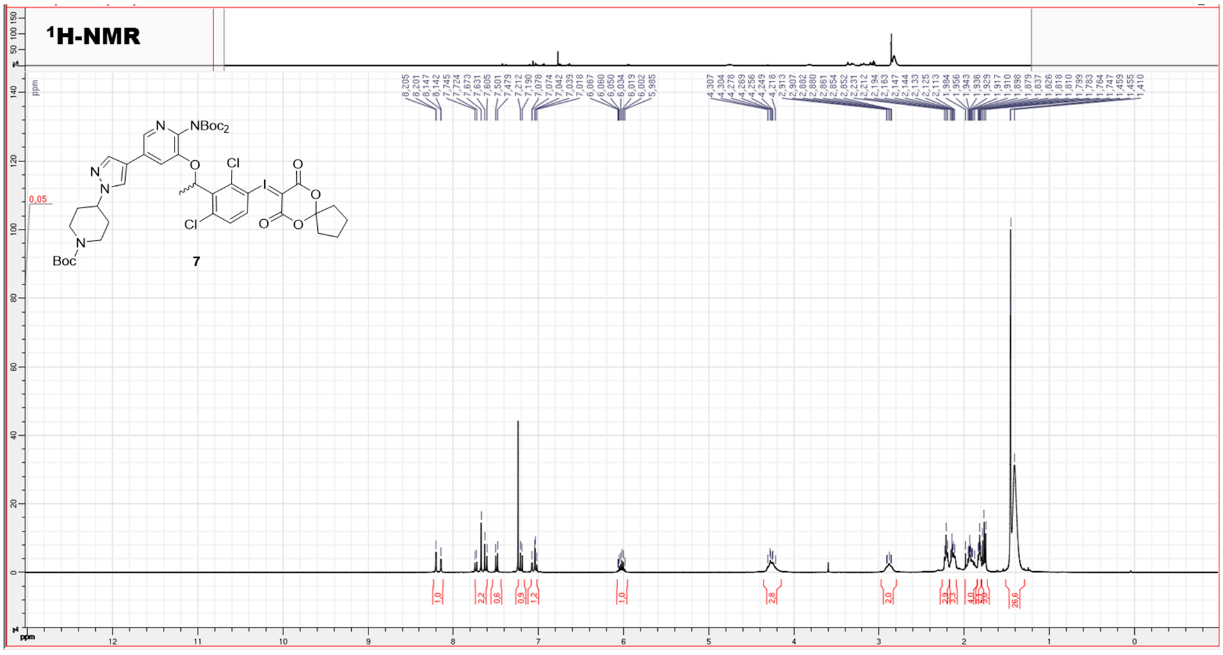

Figure S6. ^{1}H NMR of compound **7**.

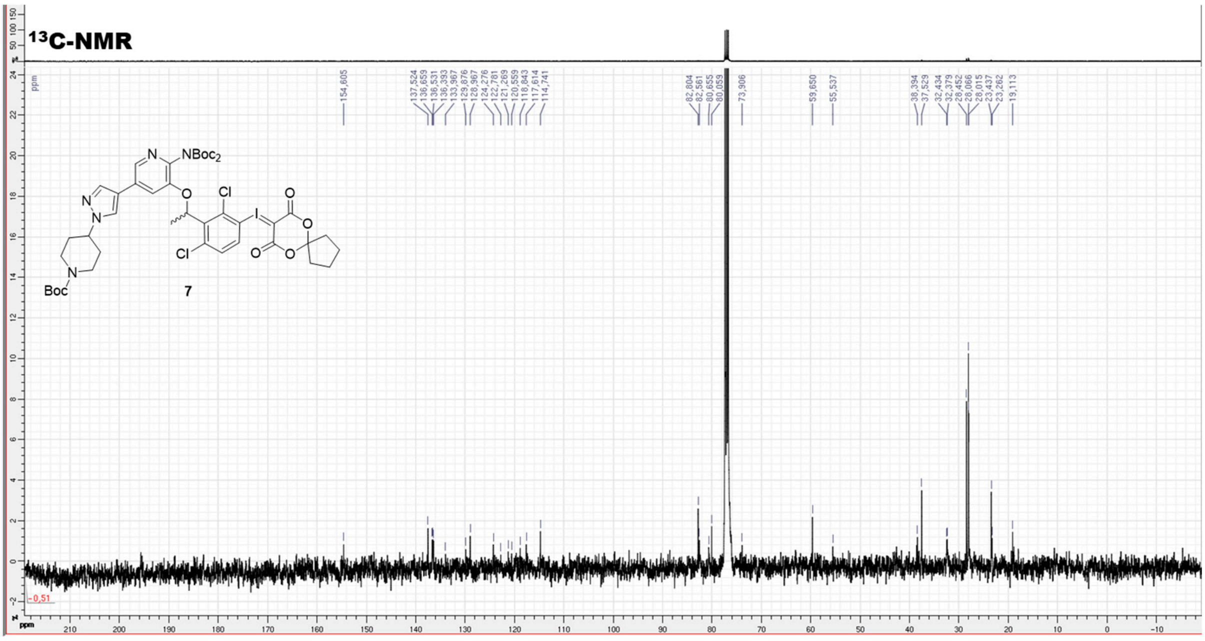

Figure S7. ^{13}C NMR of compound **7**.

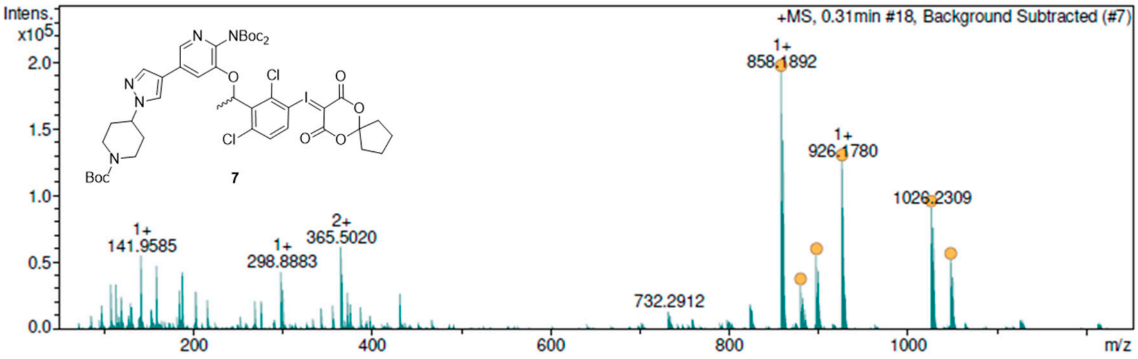

Meas. m/z	z	#	Ion Formula	m/z	err [ppm]	mSigma	rdb	e⁻ Conf
1026.230854	1+	1	C44H55Cl2IN5O11	1026.231437	0.6	28.3	19.0	even
926.177975	1+	1	C39H47Cl2IN5O9	926.179008	1.1	16.9	18.0	even

Figure S8. HRMS analysis of compound **7**.

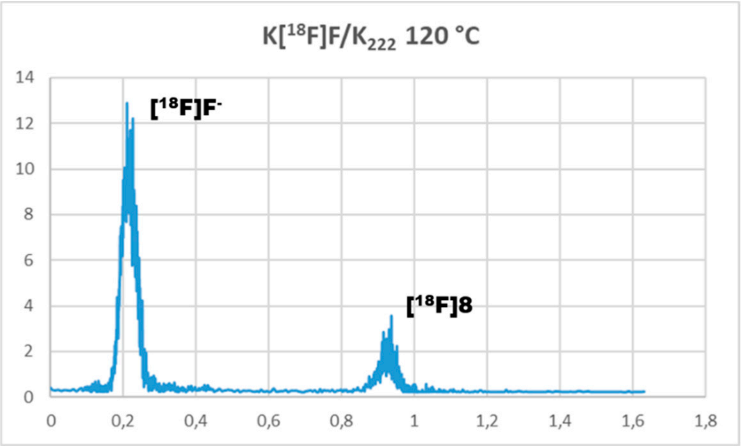

Figure S9. TLC of the crude radiofluorination of **7** using the K[^{18}F]F/K$_{222}$ complex in DMF at 120 °C for 10 minutes.

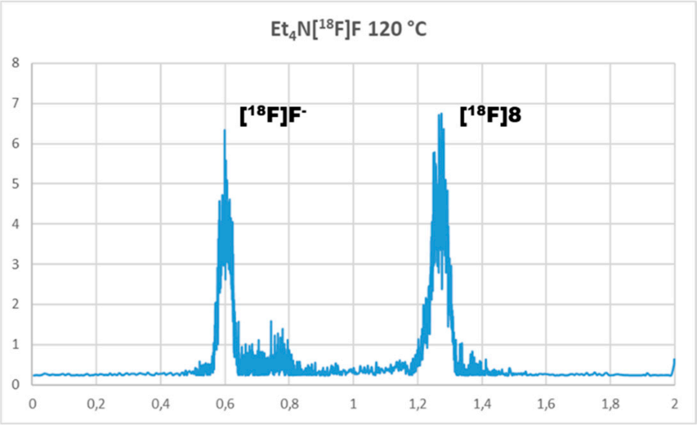

Figure S10. TLC of the crude radiofluorination of **7** using Et$_4$N[^{18}F]F in DMF at 160 °C for 10 minutes.

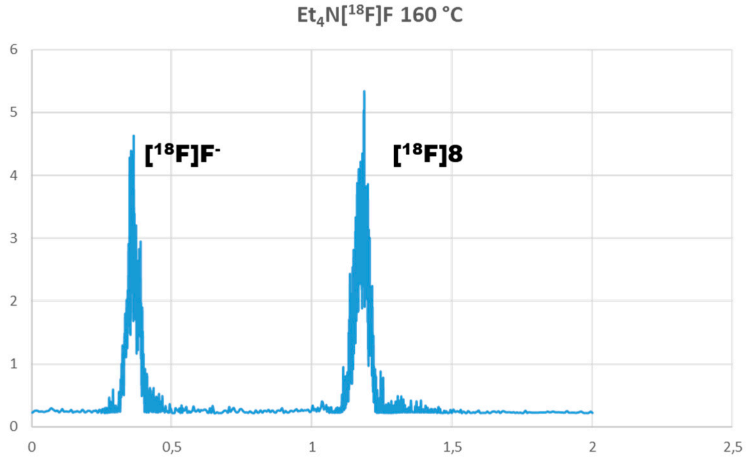

Figure S11. TLC of the crude radiofluorination of **7** using Et$_4$N[^{18}F]F in DMF at 160 °C for 10 minutes.

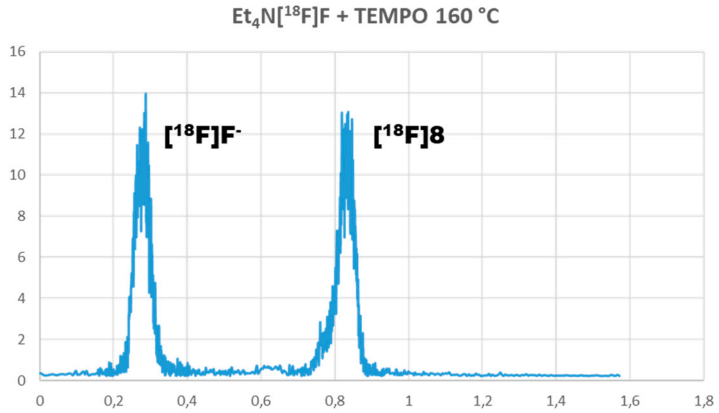

Figure S12. TLC of the crude radiofluorination of **7** using Et$_4$N[^{18}F]F and TEMPO (1 mg) in DMF at 160 °C for 10 minutes.
